# Refining computational inference of gene regulatory networks: integrating knockout data within a multi-task framework

**DOI:** 10.1093/bib/bbae361

**Published:** 2024-07-31

**Authors:** Wentao Cui, Qingqing Long, Meng Xiao, Xuezhi Wang, Guihai Feng, Xin Li, Pengfei Wang, Yuanchun Zhou

**Affiliations:** Computer Network Information Center, Chinese Academy of Sciences, CAS Informatization Plaza No. 2 Dong Sheng Nan Lu, Haidian District, Beijing, 100083, China; University of Chinese Academy of Sciences, No. 19A Yuquan Road, Shijingshan District, Beijing, 100049, China; Computer Network Information Center, Chinese Academy of Sciences, CAS Informatization Plaza No. 2 Dong Sheng Nan Lu, Haidian District, Beijing, 100083, China; Computer Network Information Center, Chinese Academy of Sciences, CAS Informatization Plaza No. 2 Dong Sheng Nan Lu, Haidian District, Beijing, 100083, China; University of Chinese Academy of Sciences, No. 19A Yuquan Road, Shijingshan District, Beijing, 100049, China; Computer Network Information Center, Chinese Academy of Sciences, CAS Informatization Plaza No. 2 Dong Sheng Nan Lu, Haidian District, Beijing, 100083, China; University of Chinese Academy of Sciences, No. 19A Yuquan Road, Shijingshan District, Beijing, 100049, China; University of Chinese Academy of Sciences, No. 19A Yuquan Road, Shijingshan District, Beijing, 100049, China; State Key Laboratory of Stem Cell and Reproductive Biology, Institute of Zoology, Chinese Academy of Sciences, 1 Beichen West Road, Chaoyang District, Beijing, 100101, China; University of Chinese Academy of Sciences, No. 19A Yuquan Road, Shijingshan District, Beijing, 100049, China; State Key Laboratory of Stem Cell and Reproductive Biology, Institute of Zoology, Chinese Academy of Sciences, 1 Beichen West Road, Chaoyang District, Beijing, 100101, China; Computer Network Information Center, Chinese Academy of Sciences, CAS Informatization Plaza No. 2 Dong Sheng Nan Lu, Haidian District, Beijing, 100083, China; University of Chinese Academy of Sciences, No. 19A Yuquan Road, Shijingshan District, Beijing, 100049, China; Computer Network Information Center, Chinese Academy of Sciences, CAS Informatization Plaza No. 2 Dong Sheng Nan Lu, Haidian District, Beijing, 100083, China; University of Chinese Academy of Sciences, No. 19A Yuquan Road, Shijingshan District, Beijing, 100049, China

**Keywords:** gene regulatory network, graph neural network, gene knockout

## Abstract

Constructing accurate gene regulatory network s (GRNs), which reflect the dynamic governing process between genes, is critical to understanding the diverse cellular process and unveiling the complexities in biological systems. With the development of computer sciences, computational-based approaches have been applied to the GRNs inference task. However, current methodologies face challenges in effectively utilizing existing topological information and prior knowledge of gene regulatory relationships, hindering the comprehensive understanding and accurate reconstruction of GRNs. In response, we propose a novel graph neural network (GNN)-based Multi-Task Learning framework for GRN reconstruction, namely MTLGRN. Specifically, we first encode the gene promoter sequences and the gene biological features and concatenate the corresponding feature representations. Then, we construct a multi-task learning framework including *GRN reconstruction*, *Gene knockout predict*, and *Gene expression matrix reconstruction*. With joint training, MTLGRN can optimize the gene latent representations by integrating gene knockout information, promoter characteristics, and other biological attributes. Extensive experimental results demonstrate superior performance compared with state-of-the-art baselines on the GRN reconstruction task, efficiently leveraging biological knowledge and comprehensively understanding the gene regulatory relationships. MTLGRN also pioneered attempts to simulate gene knockouts on bulk data by incorporating gene knockout information.

## Introduction

Understanding the intricate mechanisms that govern gene expression and regulation is crucial for unraveling the complexities of biological systems [1–3]. Gene regulatory networks (GRNs) play a pivotal role in orchestrating the dynamic interactions among genes, controlling various cellular processes, and influencing organismal development and function. Reconstructing GRNs from experimental data provides valuable insights into the underlying regulatory relationships and offers a comprehensive view of cellular behavior [4]. The continuous progress in high-throughput sequencing technology has spurred the development of numerous computational methods. These approaches employ diverse analytical strategies and modeling techniques to unravel the complexities inherent in GRN reconstruction, categorizing them broadly into three classes: statistical approaches and machine learning models, supervised deep neural networks, and graph neural networks (GNNs).

**Methods based on statistical approaches and machine learning models**. These methods utilize similarity or correlation-based techniques to identify indirect interactions from gene expression data, including linear regression [5], mutual information [6], Pearson and Spearman correlation [7], Bayesian networks [8], and Gaussian graphical models [9]. GENIE3[10] or GRNBoost2[11] employ tree-based regression to investigate genes co-expressed with transcription factors (TFs). DeepSEM[12] introduces a structural equation model with a $\beta $-VAE framework to predict the regulatory relationships between genes in the adjacent matrix of GRN. Due to the absence of supervised information, these methods may identify gene interactions rather than direct regulatory relationships. Additionally, the inference performance is significantly constrained by the input data’s quality, given the presence of missing values and high noise in gene expression.

**Methods based on supervised deep neural networks**. The core idea of such methods is to utilize known regulatory relationships as supervised information, employing deep learning techniques for GRN reconstruction [13, 14]. Compared with unsupervised learning, supervised models can identify much more subtle differences between positive and negative pairs [15]. GripDL [16] connects two graphs (originating from TFs and target genes, respectively) and utilizes a single ResNet [17] to predict the existence of regulatory interactions. ConGRI [14] employs a Siamese network [18] to learn image pairs as inputs for constructing GRNs. Li *et al*. [13] propose SDINet to integrate RNA-seq data and gene expression image data. However, these methods have some limitations. Firstly, they make pairwise predictions without considering the GRN topology, meaning that the model input only includes expression data for pairs of genes. Secondly, they are unable to predict gene pairs with missing expression data.

**Methods based on GNNs**. In recent years, the rapid development of GNN has significantly enhanced the performance of tasks involving graph-structured data [19–22]. GNNs differ from traditional approaches by learning not only from pairs of gene features but also from the entire graph, encompassing both node features and the topological information of the graph. Consequently, GNNs have emerged as a promising choice for modeling GRNs, leading to the proposal of several GNN-based GRN inference methods [13, 23, 24]. These methods frame the supervised GRN inference problem as a link prediction task: given a set of genes and their observed interactions, the goal is to predict other potential links within the network. For instance, Yuan and Bar-Joseph [23] employed graph convolutional networks (GCNs) to infer gene interactions from spatial transcriptomics data. Wang *et al*. [24] devised an integrated framework, GRGNN, which utilizes extracted subgraphs to construct GRNs. Bigness *et al*. [25] harnessed the power of GCN to predict gene expression (RNA-seq) by leveraging histone modifications and Hi-C data. Karbalayghareh *et al*. [26] introduced the GraphReg model, a novel approach that propagated local representations learned from one-dimensional inputs using graph attention network (GAT) layers over three-dimensional interaction graphs. This model effectively predicted gene expression (CAGE-SEQ) across genomic regions (BIN). GNNLink [27] utilizes a GCN-based interaction graph encoder to optimize gene features, capturing interdependencies between nodes to infer GRN. GMFGRN [28] employs GNN for matrix factorization to learn representative embeddings for genes and utilizes these embeddings to determine interactions between TF–gene pairs. However, current GNN-based methods do not effectively leverage prior biological knowledge.

Prior biological knowledge related to gene regulation plays a crucial role in the construction of GRNs [18, 29]. Gene knockout provides direct insights into gene functionality, allowing us to observe changes induced in a biological system by eliminating the expression of specific genes. By identifying which gene knockouts lead to expression changes in other genes, we can infer their direct or indirect interactions. The promoter is a key region for regulating gene expression, containing important elements such as TF binding sites [1]. Understanding the promoter sequence allows us to identify which TFs may be associated with the regulation of specific genes, aiding in revealing direct regulatory relationships within the GRN. Incorporating this prior biological knowledge enhances the accuracy and reliability of the constructed GRNs by guiding the selection of relevant features and relationships.

In this paper, we propose an innovative multi-task learning framework based on GNN for the reconstruction of GRNs. Our model effectively reconstructs GRNs by learning low-dimensional vector representations of gene expression in the presence of an incomplete prior network, leveraging and integrating prior biological knowledge related to gene regulation. Addressing the challenge of underutilizing relevant biological knowledge in GNN-based GRN reconstruction, we encode features such as promoter characteristics and multiple biological attributes related to gene regulation as node features to optimize the latent representation vectors of genes. This optimization enhances the accuracy of GRN reconstruction. To tackle the underutilization of gene knockout information in existing approaches, we download and integrate global gene knockout data, introducing a gene knockout task to effectively utilize this information; furthermore, we have undertaken attempts at predicting gene knockouts in this auxiliary task. Simultaneously, we introduce a task for reconstructing gene expression matrices to address the issue of noise in gene expression data. Our experiments demonstrate that our method achieves the best performance in the task of reconstructing GRNs. Overall, our major contributions to this work can be summarized as follows:

- We introduce a multitasking framework that seamlessly incorporates gene knockout information, directly linked to gene regulation, along with other essential prior biological knowledge during the construction of GRNs.
- Addressing the underutilization of gene knockout data, our approach integrates global gene knockout information, introducing a novel task that effectively exploits these data and significantly contributes to a more accurate reconstruction of GRNs. Notably, we pioneered attempts at simulating gene knockouts on bulk data, marking our knowledge as the first endeavor in this domain.
- MTLGRN pioneers the utilization of promoter sequence information to predict regulation patterns, marking a groundbreaking effort in integrating sequence-based insights for enhanced understanding of gene regulatory relationships.
- MTLGRN has proved to be efficient and effective for GRN construction based on the results of our experiment.

## Materials and methods

### Model framework

This problem is formulated as a link-prediction task on an undirected graph $G = (V, E, X)$. Let $V$ denote the set of TFs and genes, $E$ represents the regulatory interactions between TFs and genes, and $X\in \mathbb{R}^{m\times n}$ represent the feature matrix of the node. The goal is to predict potential edges in $E$ based on known interactions and additional biological features. Mathematically, the task involves working with the adjacency matrix $A$ of the undirected graph, where ${A}_{ij}=1$ indicates a regulatory interaction between TF $i$ and gene $j$, and ${A}_{ij}=0$ indicates no known interaction. The aim is to reconstruct an estimated adjacency matrix $\widehat{A}$, where $\widehat{A}_{ij}$ represents the predicted likelihood of interaction between TF $i$ and gene $j$.

**Figure 1:**
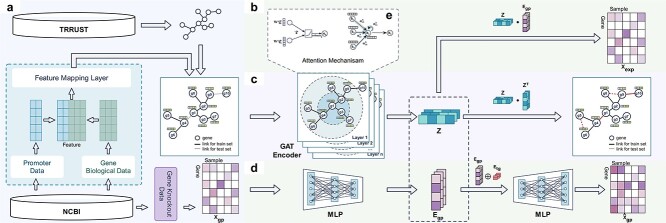
Overview of our proposed MTLGRN, which consists of three components: (a) Feature Representation Module: encodes promoter sequence features and various biological characteristics of genes; (b) Graph Autoencoder Module: reconstructs the GRN, revealing latent patterns and potential TFs and genes through an autoencoder-based approach; (c) Gene Expression Matrix Reconstruction Module: targets noise optimization in gene expression data; (d) Gene Knockout Prediction Module: integrates gene knockout data into the GRN reconstruction process, facilitating predictions on gene knockout events; (e) the demonstration of the attention mechanism in the GAT layer.

In this study, we propose a novel approach MTLGRN to predict the interactions between TFs and target genes. The MTLGRN model employs a multi-task learning framework, with the fundamental assumption that by learning data patterns from known genes, it can effectively predict potential interactions between TFs and target genes. In the GRN, TFs with similar biological features are considered more likely to regulate the same target genes. As illustrated in Fig. 1, MTLGRN is divided into four parts: (1) Feature Representation Module, responsible for encoding the promoter sequence features and various biological features of TFs and genes; (2) Graph Autoencoder Module, used to reconstruct the GRN and discover potential TFs and genes; (3) Gene Expression Matrix Reconstruction Module, aimed at optimizing noise issues in gene expression data; (4) Gene knockout Prediction Module, introducing gene knockout data into the process of GRN reconstruction and making predictions on gene knockout. The multi-task architecture enables simultaneous prediction of TF–target gene interactions and gene knockout effects, leveraging shared latent representation vectors of genes and samples to learn informative feature representations. By combining these tasks, our approach facilitates more accurate predictions and provides a comprehensive understanding of the GRN and gene knockouts.

#### Feature representation

In the feature representation stage of our method, we employ different encoding approaches to encode gene promoter sequences and gene biological features. These various feature sources were subsequently reduced in dimensionality and effectively integrated into a comprehensive feature representation, providing a foundation for a robust and nuanced analysis of the GRN.

**Endcoding gene promoter sequences**: we employ k-mer encoding [30] to capture sequence-specific information from gene promoter sequences. For a given DNA sequence $S$ and $k$, $k$-mer encoding involves breaking down the sequence into overlapping subsequences of length $k$. Mathematically, the $k$-mer encoding function $\mathcal{F}$ is defined as follows:


(1) \begin{align*} & \mathcal{F}(S, k) = [S_{1}, S_{2},..., S_{n-k+1}], \end{align*}

(2) \begin{align*} & P = \mathcal{F}(S, k), \end{align*}

where $P$ refers to the final representation vector of the gene promoter region, $S$ represents the DNA sequence, $k$ is the length of k-mers, and $S_{1}, S_{2},..., S_{n-k+1}$ represent all the subsequences of length $k$ in the DNA sequence $S$.

**Endcoding gene biological features**: we used one-hot encoding to convert categorical information, like gene families, issue specificity, into a numerical format suitable for analysis. This encoding method creates a binary vector for each category, where a value of 1 indicates the presence of the category and 0 represents its absence. Mathematically, the one-hot encoding $\epsilon _{F}$ for a categorical feature $F$ with $m$ unique categories is represented as


(3) \begin{align*}& \epsilon_{F} = [b_{1}, b_{2},...,b_{i},..., b_{m}],\end{align*}

where $b_{i}$ is a binary value indicating the presence (1) or absence (0) of the $i$th category in the given feature. To merge these encoded features into a single vector, we concatenate them. The comprehensive feature vector $B$ is formed by concatenating the one-hot encoded vectors of different biological features:


(4) \begin{align*}& B = \epsilon_{F_{1}} \oplus \epsilon_{F_{2}} \oplus... \oplus \epsilon_{F_{n}},\end{align*}

where $F_{1},F_{2},...,F_{n}$ represent distinct biological features, $\oplus $ represents the concatenation operation, and $\epsilon _{F_{n}}$ is the one-hot encoded vector for the ith feature. This concatenated vector $B$ consolidates information from various biological features for subsequent analysis.

**Dimensionality reduction and feature concatenation:** following the encoding of these diverse feature sources, we applied dimensionality reduction techniques such as principal component analysis (PCA) to reduce the dimensionality of the individual feature sets. Finally, the reduced feature representations from the different feature sources were concatenated into a unified feature vector $X$:


(5) \begin{align*}& X = D(P) \oplus D(B),\end{align*}

where $D(\cdot )$ represents the dimensionality reduction process; in this paper, we employ PCA as our chosen dimensionality reduction method. $\oplus $ represents the concatenation operation.

#### GRN reconstruction

Given an initial adjacency matrix $A$ and node feature matrix $X$, MTLGRN expects to learn a mapping function $f$ that can generate low-dimensional gene embeddings $Z$ to predict latent gene–gene interactions, 


(6) \begin{align*}& Z = f_{\phi}(A,X),\end{align*}

where $\phi $ refers to the parameters of the function.

In MTLGRN, two GAT layers are employed to learn gene representations and reconstruct regulatory interactions, as shown in Fig. 1. Each node shares a parameterized weight matrix in the first GAT layer. Subsequently, a self-attention mechanism $\tau $ computes attention coefficients, denoted as $a_{ij}$, between genes $i$ and $j$ by masking the attention matrix with observed interactions and normalizing it through the Softmax function [31, 32].


(7) \begin{align*} & att_{i j}=\tau\left(W^{T} \vec{g}_{i}, W^{T} \vec{g}_{j}\right), \end{align*}

(8) \begin{align*} & \alpha_{i j}=\operatorname{softmax}_{j \rightarrow N_{i}}=\frac{\exp \left(\operatorname{LeakyReLU}\left(\text{att}_{ij}\right)\right)}{\sum_{k \in N_{i}} \exp \left(\operatorname{LeakyReLU}\left(att_{i k}\right)\right)}, \end{align*}

where the input for gene features $\vec{g}_{i}={\vec{g}_{1},\vec{g}_{2},...,\vec{g}_{n}}$,$\vec{g}_{i} \in X$,$N_{i}$ represents the neighbor nodes of gene $i$ in the network structure; $LeakyReLU$ is a nonlinear activation function. The attention coefficient ${ att} _{i j}$ can be viewed as the regulatory strength between gene $i$ and gene $j$. We apply multi-head attention mechanisms to benefit robust node representations. We concatenate the $K$ independent attentions to get the output gene representations of this layer:


(9) \begin{align*}& \vec{g}_{i}^{1}=\oplus_{k=1}^{K} \sigma\left(\sum_{j \in N_{i}} \alpha_{i j}^{k} W_{k}^{T} \vec{g}_{j}\right),\end{align*}

where $\oplus $ is concatenation operation, $\alpha _{i j}^{k}$ is the kth normalized attention coefficient, $W_{k}^{T}$ is the transposed weight matrix for linear transformation, and $\sigma $ is $elu$ (exponential linear unit) function. In the second GAT layer, we average the multi-head attention by


(10) \begin{align*}& Z = {\overrightarrow{g}}_{i}^{2}=\sigma\left(\frac{1}{K} \sum_{k=1}^{K} \sum_{j \in N_{i}} \alpha_{i j}^{k} W_{k}^{T} \vec{g}_{j}\right).\end{align*}

Finally, we use the dot product as the score function to evaluate the similarity of this pair of genes based on learned representations:


(11) \begin{align*}& \widehat{A} = \delta(ZZ^{T}),\end{align*}

where $\delta $ is the dot product operation. We employ binary cross-entropy loss for GRN reconstruction:


(12) \begin{align*}& \mathcal{L}_{\text{GRN}} = -\sum \left (A \cdot \log(\widehat{A}) + (1 - A) \cdot \log(1 - \widehat{A})\right ),\end{align*}

where $ A $ is the true interaction between genes, and $\widehat{A}$ is the predicted interaction score between genes.

#### Gene knockout prediction

Gene knockout prediction adds a critical perspective to our methodology, enabling researchers to predict how specific gene knockouts will influence the broader GRN. This task is instrumental in understanding the cascading effects of gene manipulation, as it reveals how knockouts in one gene can lead to changes in the expression of other genes[33]. By jointly optimizing these tasks, we gain a more comprehensive and holistic view of transcriptional regulation and gene knockouts within biological systems.

In the context of gene knockout prediction, we employ a multi-layer perceptron (MLP) encoder. This encoder processes the gene expression data before the knockout of the target gene and transforms them into a lower dimensional vector representation. The mathematical representation of this encoder is as follows:


(13) \begin{align*}& E_{\text{gp}} = \sigma(W_{\text{enc}} X_{\text{gp}} + b_{\text{enc}}),\end{align*}

where $E_{\text{gp}}$ represents the encoded gene expression data, $W_{\text{enc}}$ and $ b_{\text{encoder}}$ are the weight matrix and bias of the encoder, respectively, $X_{\text{g}}$ is the input gene expression data, and $\sigma $ is the activation function.

To predict the effects of gene knockout, the encoded vector $E_{\text{gp}}$ is concatenated with another vector representing the target gene $E_{\text{tg}}$ to be knocked out:


(14) \begin{align*}& E_{\text{con}} = E_{\text{gp}} \oplus E_{\text{tg}},\end{align*}

where the $E_{\text{tg}}$ is derived from the knockout data preprocessed with one-hot vector conversion and dimensionality reduction methods. The concatenated vector $ E_{\text{con}}$ is then processed through an MLP decoder to generate the gene expression data after the knockout:


(15) \begin{align*}& \widetilde{X}_{\text{gp}}= \sigma(W_{\text{dec}} \cdot E_{\text{con}} + b_{\text{dec}}),\end{align*}

where $\widetilde{X}_{\text{gp}}$ represents the predicted gene expression data after the knockout, and $W_{\text{dec}}$ and $b_{\text{dec}}$ are the weight matrix and bias of the decoder, respectively.

The loss function for the gene knockout prediction task is a mean squared error (MSE) loss [34], which measures the dissimilarity between the predicted gene expression data after knockout $\widetilde{X}_{\text{gp}}$ and the actual gene expression data after the knockout $X_{\text{gp}}$:


(16) \begin{align*}& L_{perturb} = \frac{1}{N} \sum_{i=1}^{N} \left(X_{\text{gp}}^{i} - \widetilde{X}_{\text{gp}}^{i} \right)^{2},\end{align*}

where $N$ represents the total number of genes in the dataset, and $i$ denotes an individual gene.

#### Gene expression matrix reconstruction

Gene expression data are often subject to noise and technical limitations, leading to the presence of missing or inaccurate data points. Therefore, the method of reconstructing gene expression matrices becomes particularly significant as it aims to fill these gaps and rectify damaged data, thereby enhancing data integrity and reliability. Furthermore, by amalgamating diverse sources of biological data, such as GRNs and gene expression data, this approach provides a more comprehensive information landscape for explaining gene expression variations. This aids biologists in better comprehending the interplay between GRNs and gene expression, thereby shedding light on the mechanisms of biological phenomena.

We multiply the obtained gene representation vectors and sample representation vectors to reconstruct the gene expression matrix. The reconstruction of the gene expression matrix is defined as


(17) \begin{align*}& X_{exp} = \delta(E_{\text{gp}} Z),\end{align*}

where $\delta $ is dot product operation, $X_{exp}$ represents the reconstructed gene expression matrix, $E_{\text{gp}}$ stands for the gene representation matrix, and $Z$ denotes the sample representation matrix.

To train the model, we define the reconstruction error of the gene expression matrix as the loss function, expressed as


(18) \begin{align*}& L_{\text{exp}} = \frac{1}{N}\sum_{i=1}^{N}\sum_{j=1}^{M}(X_{ij} - X_{\text{exp}_{ij}})^{2},\end{align*}

where $N$ is the number of samples, $M$ is the number of genes, $X_{ij}$ represents the element in the original gene expression matrix, and $X_{exp_{ij}}$ signifies the element in the reconstructed gene expression matrix.

#### Joint training

Similar to the overall model architecture, the Gene knockout prediction task is jointly optimized with the GRN reconstruction and Gene expression matrix reconstruction. A unified loss function combines the binary cross-entropy loss for GRN reconstruction $\mathcal{L}_{\text{GRN}}$ with the MSE loss for both gene expression matrix reconstruction $\mathcal{L}_{\text{exp}}$ and gene knockout prediction $\mathcal{L}_{\text{perturb}}$:


(19) \begin{align*}& L_{\text{joint}} = \lambda_{1} \cdot L_{\text{GRN}} + \lambda_{2} \cdot L_{\text{perturb}} + (1-\lambda_{1}-\lambda_{2}) \cdot L_{\text{exp}},\end{align*}

where $\lambda _{1},\lambda _{2}$ is a hyperparameter used to balance the relative importance of the three loss functions: $L_{\text{GRN}}$, $L_{\text{perturb}}$, and $L_{\text{exp}}$. Such hyperparameters can be tuned based on the specific requirements of the task and the characteristics of the dataset, aiming to achieve a better overall balance in the model’s performance.

## Experimental and results

### Dataset

In constructing our initial GRN, we utilized the TRRUST database (https://www.grnpedia.org/trrust/) [35], documenting 6490 regulatory connections between 827 mouse TFs and 1629 target genes. The database, compiled through advanced text mining from 11 237 PubMed articles, characterizes TF–target gene pairs by activation, repression, or unknown regulatory modes. For streamlined analysis, we grouped interactions as “activation,” “repression,” or “unknown” into a single category termed “interactive.” This approach allows us to focus on identifying the existence of TF–target interactions without predicting specific types. Notably, interactions with both “activation” and “repression” annotations are managed by classifying them as redundant, maintaining a singular link for each occurrence.

Promoter sequence features, spanning 2.5 kilobase pairs and sourced from NCBI, play a crucial role in understanding gene regulation. These features undergo meticulous preprocessing, including sequence pattern analysis and motif identification, before integration into our dataset. Their incorporation enriches the accuracy of our network reconstruction, providing essential insights into the regulatory dynamics of genes within the network.

Diverse gene biological features(The detailed description can be found in Table S1), such as protein-coding status and TF activity, gene families, contribute to our comprehensive understanding of gene regulation. Sourced from various databases and literature, these features undergo thorough processing to ensure data reliability before integration. Their inclusion enhances our grasp of gene–gene interactions and regulatory mechanisms, enriching the overall structure and dynamics of the GRN.

Gene knockout data, acquired globally from NCBI GEO, undergo a rigorous selection process and comprehensive bulk RNA-seq pipeline analysis. Steps include quality control assessment, adapter trimming, and read alignment to a reference genome. This meticulous processing ensures the reliability of the gene knockout data before its integration into our dataset. The systematic acquisition and analysis of these datasets significantly contribute to our comprehensive network reconstruction, providing insights into disruptions caused by the specific absence of genes and their impact on biological processes.

### Baseline

The performance of MTLGRN is compared with four classical machine learning methods including GENIE3 [10], GRNBoost2 [11], SCENIC [36], and STGRNS [37], and three GNN-based methods including DeepWalk [38], GraphTGI [39], and DeepTFni [13].

- **GENIE3**: It utilizes Random Forests to rank potential regulators for each gene based on gene expression data, constructing a global network.
- **GRNBoost2**: It employs a gradient-boosting framework to model relationships between genes, identifying direct regulatory interactions.
- **SCENIC**: It integrates single-cell RNA sequencing data with TF binding motifs to infer GRNs and identify cell states.
- STGRNS: It utilizes interpretable transformer-based methodology to enhance the accuracy of inferring GRNs from scRNA-seq data.
- **DeepWalk**: It utilizes random walks on the graph and deep learning techniques to learn continuous vector representations for nodes, commonly used for node classification and link prediction.
- **GraphTGI**: It leverages a GAT to model interactions between genes and identify regulatory relationships within the network.
- **DeepTFni**: It harnesses the capabilities of Variational Graph Autoencoder to infer GRNs from single-cell assays that assess transposase-accessible chromatin through sequencing (scATAC-seq) datasets.

### Evaluation criteria

We used GENIE3, GRNBoost2, SCENIC, STGRNS, DeepWalk, GraphTGI, and DeepTFni source code with tailored inputs. Adopting a standardized weighted matrix representation, we binarized the outputs for a fair comparison. By running the experiments five times on the mouse dataset, we filtered links labeled “1” in fewer than three binarized matrices to infer the potential interactions between genes. The evaluation metrics include accuracy, precision, recall, F1-Score, and AUROC, and the average value of multiple experiments is reported. The lack of non-interaction samples was validated with experimental data, hindering the construction of the negative sample set. Hence, we adopt a negative sampling strategy to construct the corresponding “negative” sample set by randomly sampling from unlabeled pairs as the positive pairs rarely appear in the unlabeled set.

### Overall performance

The experimental results, as summarized in Table 1, demonstrate the superior performance of our proposed method compared with state-of-the-art approaches. Notably, our method achieves an impressive accuracy of 0.911, outperforming DeepTFni, the closest competitor, by a substantial margin. This highlights the efficacy of our approach in accurately reconstructing GRNs. In terms of AUROC, precision, recall, and F1-score, our method consistently outperforms other methods. The AUC of 0.938 signifies the robust discriminatory power of our model, while the precision, recall, and F1-score values of 0.891, 0.887, and 0.884, respectively, demonstrate a high-performance predictive capability on the GRN construction task. Compared with DeepWalk, GENIE3, GRNBoost2, SCENIC, and STGRNS, MTLGRN achieves absolute improvements of at least 3.9%, 2%, 3%, 2%, and 2.8% in accuracy, AUROC, precision, recall, and F1-score, respectively. This success can be attributed to the strategic integration of gene features and sequence information, coupled with the incorporation of gene knockout data and the synergy achieved through multi-task learning.

**Table 1:** Performance comparison of different methods

Method	Accuracy	AUROC	Precision	Recall	F1-score
GENIE3	0.562	0.583	0.442	0.472	0.457
GRNBoost2	0.545	0.543	0.471	0.487	0.479
SCENIC	0.521	0.514	0.438	0.415	0.426
STGRNS	0.680	0.829	0.714	0.683	0.698
DeepWalk	0.687	0.836	0.602	0.588	0.595
GraphTGI	0.791	0.887	0.803	0.795	0.799
DeepTFni	0.872	0.915	0.860	0.862	0.861
**MTLGRN**	**0.911**	**0.938**	**0.891**	**0.887**	**0.889**

### Study of gene knockout prediction

**Figure 2:**
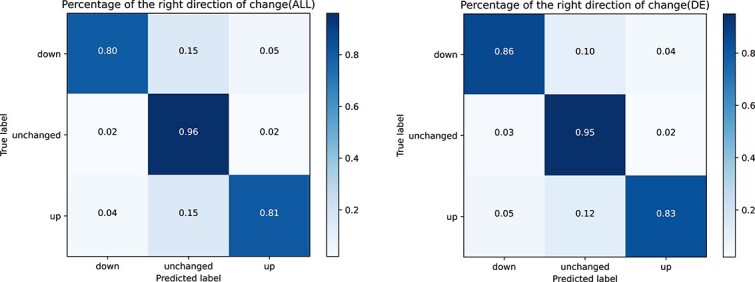
Results of right direction of change.

To assess the accuracy of gene knockout predictions, we utilized Pearson correlation coefficient (corr), MSE, and the directionality of the correct change to gauge the relationship between predicted gene expressions and actual true expression values. Additionally, we introduced a modified version of the Pearson metric, denoted as corr_$\Delta $, which quantifies the correlation between gene expression changes post-knockout and under control conditions. MSE provided a measure of the average squared differences between predicted and observed knockout phenotypes, offering deeper insights into the accuracy of our predictions in quantifying phenotypic changes. Furthermore, evaluating the correct direction of change involved examining whether our predictions captured the directionality of gene knockout effects accurately compared with experimental observations. This metric is particularly crucial as it elucidates the biological relevance of our predictions, beyond mere correlation. We reported performance comparisons with the MLP baseline for all genes (ALL) and the top 20 differentially expressed genes (DE). Our study results, as shown in Table 2, demonstrate that our method outperformed in all evaluation metrics. Additionally, Fig. 2 illustrates that our method achieved high accuracy in assessing the directionality of changes.

**Table 2:** Results of knockout prediction

Model	DE			ALL		
	corr($\uparrow $)	corr_$\Delta x$($\uparrow $)	MSE($\downarrow $)	corr($\uparrow $)	corr_$\Delta x$($\uparrow $)	MSE($\downarrow $)
MLP	0.909	0.428	0.032	0.924	0.408	0.038
**MTLGRN**	**0.938**	**0.592**	**0.015**	**0.941**	**0.579**	**0.013**

### False negatives analysis

**Figure 3:**
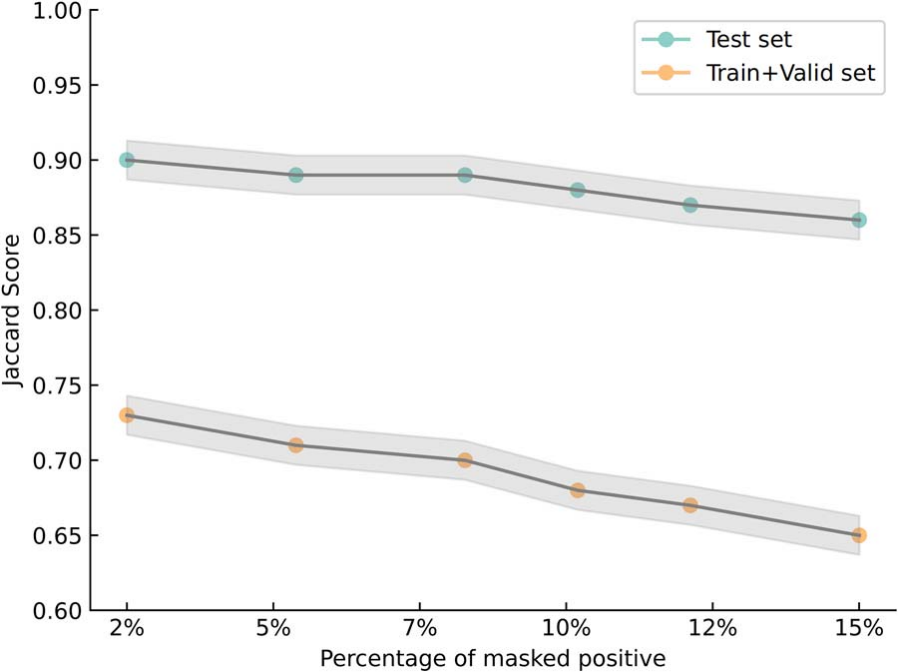
Jaccard index of interactions before and after masked-positive disturbance; the green line indicates the Jaccard index of MTLGRN prediction results on the test set; the orange line indicates the Jaccard index of disturbed train and validate set.

**Figure 4:**
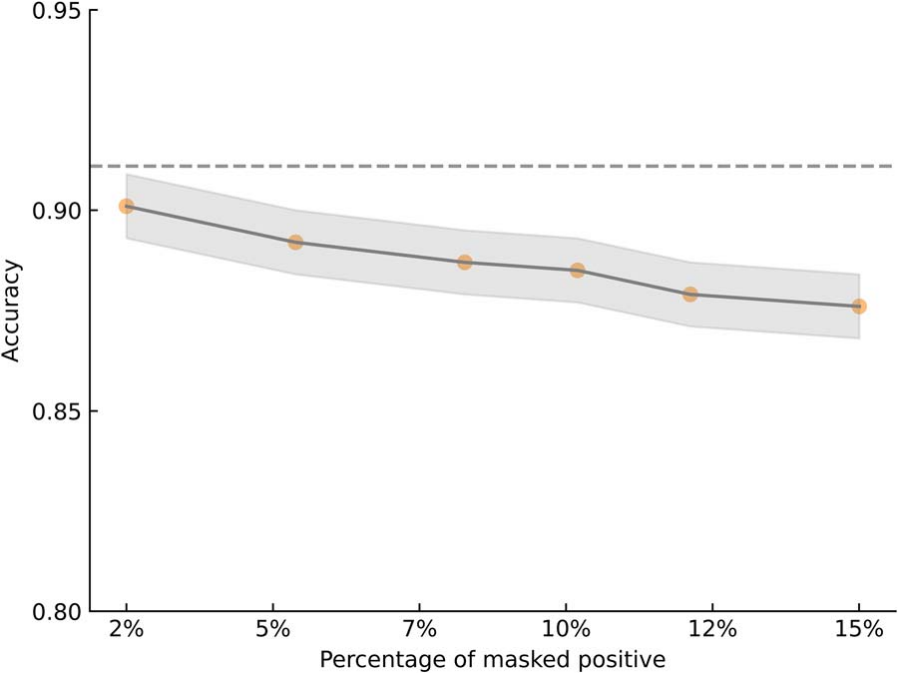
Accuracy of MTLGRN prediction in the disturbed dataset with different masked-positives proportion; the dashed line represents the accuracy without disturbance.

Based on the initial GRN data from the TRRUST database, we generated six perturbed datasets by masking 2%, 5%, 8%, 10%, 12%, and 15% of the known positive labels. The masked positives are randomly distributed throughout the dataset, simulating real-world scenarios where missing interactions are unknown. For each percentage of masked positives, we conducted the MTLGRN on its perturbed data 10 times, maintaining an identical data split as the unperturbed dataset. During training, we treated the masked positives as negatives. After the model completed training, we examined the predicted results on the test set using complete label information. As illustrated in Fig. 3, we calculated the Jaccard Index by comparing the interactions predicted between genes before and after perturbation. The results are presented in Fig. 4. During training, considering masked positives as negatives may impact the model’s learning of positive interactions on the training and validation sets. However, the observed high Jaccard index on the test set underscores the model’s robustness in predicting interactions involving unknown positives, showcasing its ability to generalize effectively to unseen positive samples. These findings suggest that the model maintains strong predictive performance even when confronted with partially hidden positive labels, demonstrating its utility in scenarios with incomplete or uncertain interaction information.

## Ablation study

### Different feature construction strategies

In this study, attributes of TF nodes and target gene nodes are incorporated into the graph of known gene interactions to enhance predictions. To assess the impact of node attribute information on our model, we compared the performance of five feature construction strategies: (i) Random node features, where features are randomized from a normal distribution; (ii) Promoter features, using one-hot vectors derived from DNA sequences as node features; (iii) Gene biological attribute features, using one-hot vectors derived from attribute features as node features; (iv) Concatenated features, where gene DNA sequence features and attribute features are concatenated to form node features.

**Figure 5:**
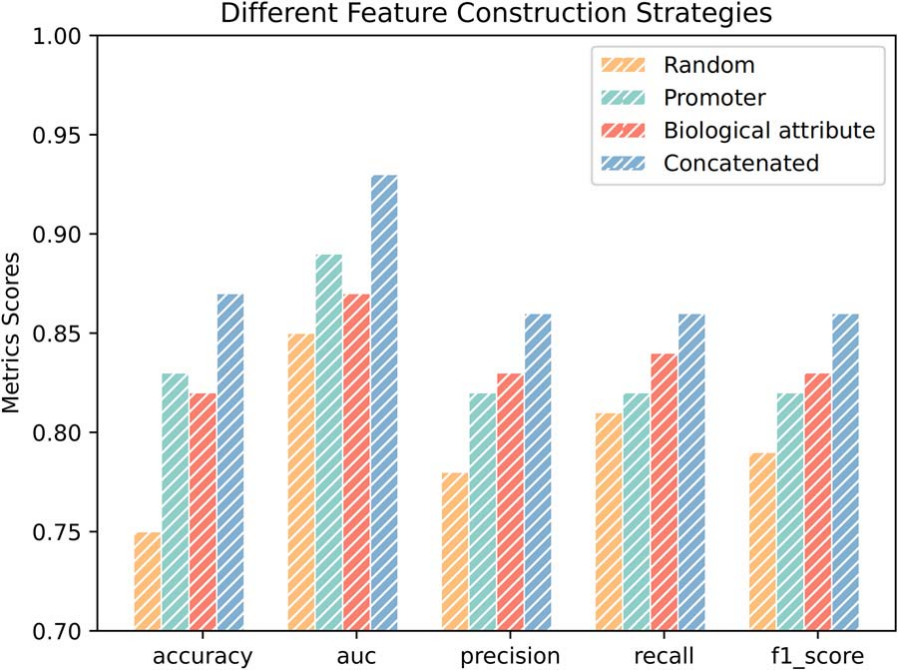
Performances comparison on the prediction of MTLGRN which use different features.

Our study uncovered performance variations in feature construction strategies for predicting TF-regulated target genes. As shown in Fig. 5, The “Concatenated Features” strategy, merging promoter and gene biological attribute features, achieved the highest accuracy, highlighting the advantage of a comprehensive node representation. Conversely, the “Random features” strategy, using randomly generated features, showed the lowest accuracy, revealing the challenge of learning meaningful patterns without structured information. The “Promoter features” and “Biological attribute Features” strategies, leveraging promoter sequence and biological attribute information, displayed intermediate accuracies, illustrating a nuanced trade-off. The superior performance of the “Concatenated Features” strategy emphasizes the complementarity of DNA sequence and attribute features, offering valuable insights into optimizing feature construction strategies for enhanced predictive models in transcriptional regulation networks.

### Different task strategies

In this section, we conduct the ablation experiments to investigate how different tasks of MTLGRN contribute to its performance. We introduce three variants as below: (i) MTLGRN w GRNR: It only retained the task of reconstructing GRNs. (ii) MTLGRN w/o GEMR: It removed the task of gene expression matrix reconstruction. (iii) MTLGRN w/o GKP: It removed the task of gene knockout prediction.

**Figure 6:**
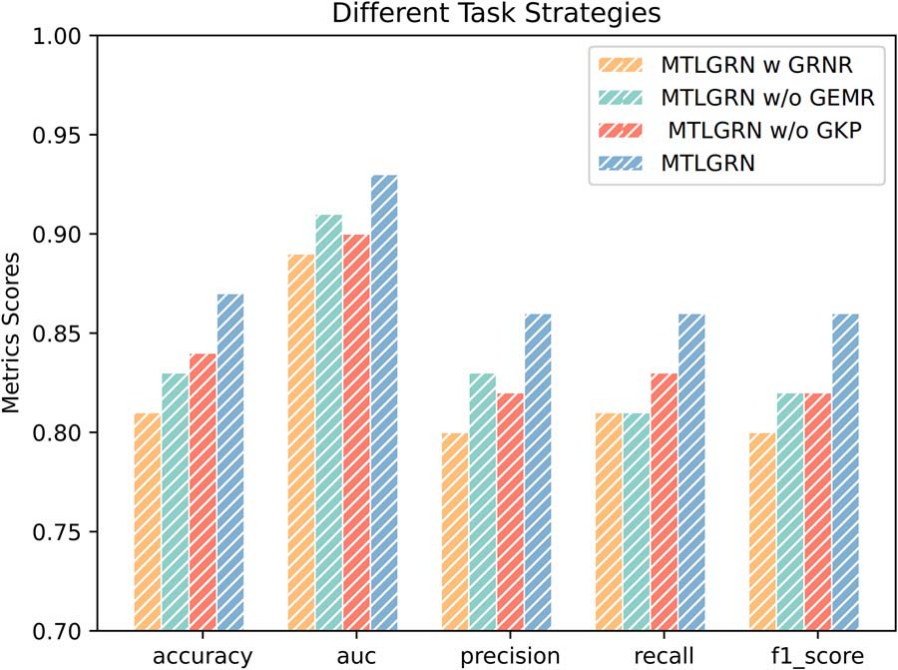
Performances comparison on the prediction of MTLGRN on different tasks.

The results depicted in Fig. 6 reveal a clear advantage in joint training. The multitasking model performs better than models trained individually for each task, indicating that it effectively captures the interconnected nature of GRNs. This enhancement suggests that simultaneously learning to reconstruct the network, gene expression matrix, and predict gene knockout outcomes leads to improved performance. These outcomes confirm the effectiveness of multitasking in GRN modeling and highlight how different tasks can complement each other during joint training. This study emphasizes the potential of multitasking approaches in understanding complex GRNs and their usefulness in computational biology. It also encourages further exploration and utilization of multitasking strategies in studying intricate biological phenomena.

## Case studies

**Figure 7:**
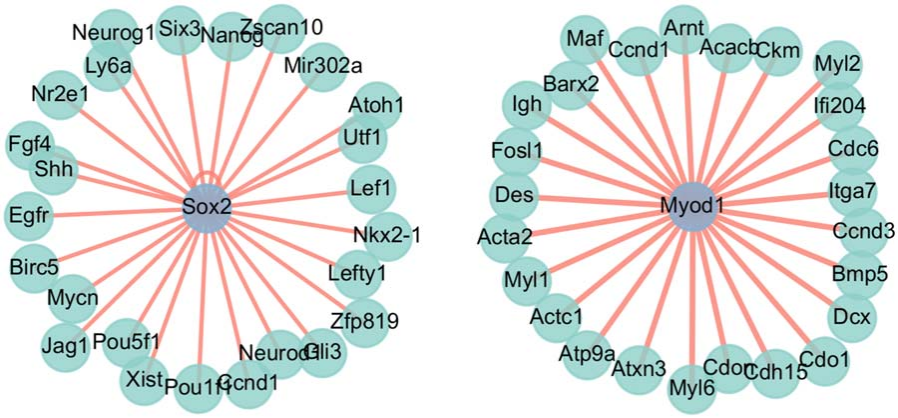
Top 20 target genes for Sox2 and Myod1 predicted by MTLGRN model.

In our exploration of mouse gene regulation, we first identified the top 10 TFs based on their degree centrality, betweenness centrality, and eigenvector centrality, as shown in Table 3. These 10 TFs are all pivotal in the regulation process of mice. Subsequently, we directed our focus toward two critical TFs: Sox2 and Myod1. In the mouse gene regulation process, Sox2 plays a crucial role in maintaining pluripotency and self-renewal, while Myod1 functions as a key regulator in promoting muscle cell differentiation. Specifically, we utilized all known TF–target gene pairs collected from the TRRUST database as the training set to train the MTLGRN model. Our focus was solely on the top-ranking predictions for the TF of interest. The subsequent validation process against ChIP-Seq data from ChIP-Atlas [40] demonstrated a robust confirmation rate of 84% for Sox2 (21/25) and an impressive 80% for Myod1 (20/25), as shown in Fig. 7. The results indicate that our model’s predictions are effective and demonstrate good generalization capabilities. Given that the top-ranked predictions are based on gene embeddings generated by MTLGRN, this finding validates that the learned gene embeddings can describe regulatory networks, revealing potential interactions between TFs and target genes.

**Table 3:** Gene centrality measures in GRN

**Gene**	**Degree centrality**	**Betweenness centrality**	**Eigenvector centrality**
Sox2	0.84	0.57	0.68
Nanog	0.83	0.52	0.67
Myod1	0.82	0.52	0.65
Oct4	0.80	0.51	0.67
Pax6	0.79	0.45	0.63
Tcf3	0.78	0.44	0.66
Foxp3	0.76	0.47	0.61
Hoxa9	0.75	0.45	0.62
Klf4	0.73	0.41	0.58
Nkx2-5	0.72	0.38	0.54

## Discussion and conclusion

Unraveling the intricacies of GRNs is pivotal for deciphering the complexities inherent in biological systems. Our proposed framework leverages GNNs for GRN reconstruction, addressing the challenge of underutilizing prior biological knowledge. Specifically, we integrate promoter characteristics and various biological attributes related to gene regulation as node features, optimizing gene representation vectors. This incorporation enhances the accuracy of GRN reconstruction by guiding the selection of relevant features and relationships.

To tackle the underutilization of gene knockout data, a critical source of direct insights into gene functionality, our method integrates global gene knockout information. This includes a novel task focused on effectively utilizing these data, pioneering simulated gene knockouts on bulk data—an unprecedented contribution in this domain. Simultaneously, we introduce a task for reconstructing gene expression matrices to mitigate noise in gene expression data, ensuring the robustness of our approach. Our experiments demonstrate the superior performance of our method in reconstructing GRNs.

However, there are still areas for improvement and future research directions. Addressing issues with data quality and availability, including noise and biases, is crucial for enhancing GRN reconstruction reliability. Additionally, integrating more omics data, such as epigenetic information and protein–protein interaction networks, could enrich the model’s predictive power and deepen our understanding of gene regulatory mechanisms.

In conclusion, our contributions lie in the development of an efficient and effective multi-task learning framework that leverages GNNs, effectively incorporates prior biological knowledge, addresses the underutilization of gene knockout data, and pioneers the use of promoter sequence information for enhanced GRN reconstruction. This strategic combination not only enhances the accuracy and interpretability of GRN modeling but also establishes a powerful platform for predicting the effects of gene knockouts, contributing to a deeper understanding of regulatory mechanisms.

**Key Points**

- MTLGRN introduces a multitasking learning framework that incorporates gene knockout information and various prior biological knowledge, improving the accuracy of GRN reconstruction.
- We systematically processed global bulk gene knockout data and conducted predictive experiments for gene knockouts on these datasets as a subtask.
- MTLGRN pioneers the utilization of promoter sequence information to predict regulation patterns.
- This work presents a new solution for integrating prior knowledge into the process of reconstructing GRNS.

## Supplementary Material

BIB24_GRN_Supplementary_bbae361

## Data Availability

The data and source code used in MTLGRN are available at https://github.com/wentaoStyle/MTLGRN.
