# Supplementary material for: Refining computational inference of gene regulatory networks: integrating knockout data within a multi-task framework
Source: Brief Bioinform. 2024 Jul 31;25(5):bbae361. doi: 10.1093/bib/bbae361 (PMC11289685; doi:10.1093/bib/bbae361)
Supplement: BIB24_GRN_Supplementary_bbae361 [file bib24_grn_supplementary_bbae361.pdf]

Supplementary material for "Refining Computational Inference of Gene Regulatory Networks: Integrating Knockout Data within a Multi-Task Framework"

Supplementary 1. Detailed introduction to biological feature

Table S1 provides detailed descriptions of biological knowledge features. In our gene regulatory network(GRN) reconstruction process, we integrate known biological knowledge into the analysis by encoding it into feature vectors. This encoding is achieved using a one-hot encoding scheme, where each known biological feature is represented as a binary vector. For example, if a gene belongs to a specific biological feature, such as a gene family, the corresponding position in its vector is set to 1; otherwise, it remains 0. This approach allows us to represent various biological features, including protein-coding genes, transcription factors, RNA-binding proteins, DNA-binding proteins, gene families, essential genes, tissue-specific genes, epigenetic modifiers, and epigenetic factors.

Given the extensive nature of biological knowledge, especially with 1500 gene families involved, the resulting feature vectors can become quite lengthy. To mitigate this, we employ dimensionality reduction techniques, such as Principal Component Analysis (PCA), to reduce the dimensionality of the feature space while preserving its essential characteristics. By applying PCA, we transform the high-dimensional feature vectors into lower-dimensional representations, which capture the most significant variation in the data. Ultimately, the resulting feature vectors, derived from the amalgamation of various biological knowledge features and reduced in dimensionality through PCA, serve as comprehensive representations of the biological knowledge incorporated into our GRN reconstruction process.

Supplementary 2. Parameter sensitivity

Sensitivity analysis is crucial for understanding how changes in hyperparameters affect MTLGRN's performance. In particular, $\lambda 1$ and $\lambda 2$ balance the $\mathcal{L}_{\text{GRN}}$, $\mathcal{L}_{\text{exp}}$ and $\mathcal{L}_{\text{perturb}}$. Therefore, we vary $\lambda 1$ and $\lambda 2$ by $\{0.2, 0.4, 0.6, 0.8, 1.0\}$ to find their optimal values. According to Figure S1, the near optimal comprehensive performance at $\lambda 1 = 0.6$ and $\lambda 2 = 0.2$ justifies our parameter settings.

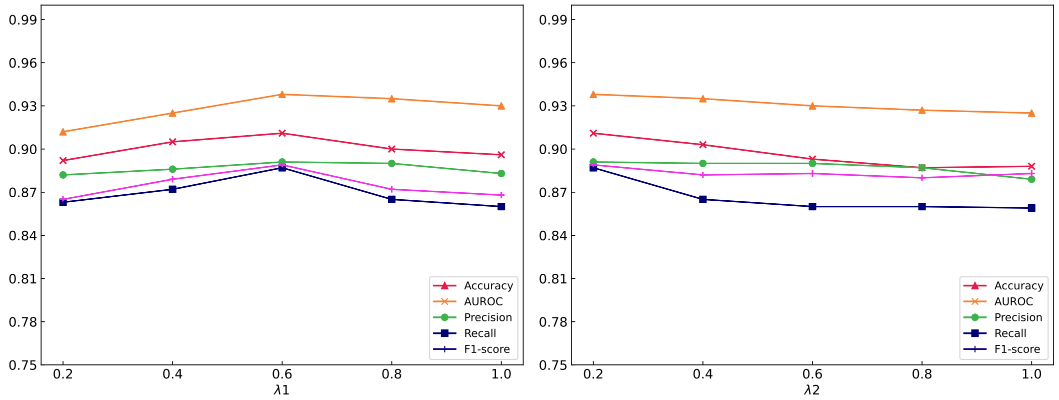

Figure S1: Paramater sensitivity analysis

Supplementary 3. Average running time of different methods

Table S2 provides insights into the computational efficiency of various methods for inferring gene regulatory networks. Traditional methods like GENIE3 and GRNBoost2 require longer average running times, exceeding 10 minutes. In contrast, graph neural network-based approaches, such as DeepWalk and GraphTGI, significantly reduce computational overhead, completing analyses in seconds rather than minutes. Our proposed method, MTLGRN, stands out with the shortest average running time of 14.67 seconds, emphasizing its efficiency in network inference tasks.

Table S1: Biological feature description

**Feature**	**Feature Type**	**Reference Database**	**Description**
Protein_coding or lncRNA	Gene type	Ensembl	Indicates whether the gene is a protein-coding gene or a long non-coding RNA (lncRNA) gene.
TF_transcription_factor	Gene type	TRRUST v2.0	Transcription factors (TFs) are proteins that bind to specific DNA sequences and regulate gene expression.
RNA_binding protein	Gene type	EuRBPDB	Genes encoding RNA-binding proteins interact with RNA and participate in post-transcriptional regulatory processes.
DNA_binding protein	Gene type	ENPD	Genes encoding DNA-binding proteins interact with DNA and regulate gene expression and transcription.
Gene_family	Gene type	HGNC	Genes are grouped into families based on sequence and functional similarities.
Essential_gene	Gene type	DEG database	Essential genes control fundamental cellular processes and their knockout may result in cell death.
Tissue_specific_gene	Gene type	GTEx	Tissue-specific genes exhibit specific expression patterns in different tissues.
Epigenetic_modifiers	Gene type	dbEM	Genes involved in regulating heritable changes in gene function without altering the DNA sequence.
Epigenetic_factors	Gene type	Epifactors	Genes involved in epigenetic regulation, including histones and protamines.

Table S2: Average running time of different methods

Method	Average running time
GENIE3	10 min 27 s
GRNBoost2	13 min 53 s
SCENIC	6 min 47 s
STGRNS	8 min 30 s
DeepWalk	42.38 s
GraphTGI	15.19 s
DeepTFni	21.58 s
**MTLGRN**	**14.67 s**
